# Interactional competencies in medical student admission– what makes a “good medical doctor”?

**DOI:** 10.1007/s10459-024-10348-w

**Published:** 2024-07-15

**Authors:** Leonie Fleck, Dorothee Amelung, Anna Fuchs, Benjamin Mayer, Malvin Escher, Lena Listunova, Jobst-Hendrik Schultz, Andreas Möltner, Clara Schütte, Tim Wittenberg, Isabella Schneider, Sabine C. Herpertz

**Affiliations:** 1 Medical Faculty, Heidelberg University, Heidelberg, Germany; 2 Central Institute of Mental Health, Mannheim, Germany; 3 Department of Child & Adolescent Psychiatry, University of Heidelberg, Heidelberg, Germany; 4 Department of General Internal Medicine and Psychosomatics, University of Heidelberg, Heidelberg, Germany; 5 Department of General Psychiatry, Heidelberg University Hospital, Voßstraße 4, 69115 Heidelberg, Germany

**Keywords:** Communication, Interaction, Admission to medical schools, Emotional availability, Doctor-patient relationship

## Abstract

Doctors’ interactional competencies play a crucial role in patient satisfaction, well-being, and compliance. Accordingly, it is in medical schools’ interest to select candidates with strong interactional abilities. While Multiple Mini Interviews (MMIs) provide a useful context to assess such abilities, the evaluation of candidate performance during MMIs is not always based on a solid theoretical framework. The newly developed selection procedure “Interactional Competencies – Medical Doctors (IC-MD)” uses an MMI circuit with five simulation patient scenarios and is rated based on the theoretically and empirically grounded construct of emotional availability. A first validation study with *N* = 70 first-semester medical students took place in 2021. In terms of convergent validity, IC-MD ratings showed strong correlations with simulation patients’ satisfaction with the encounter (*r* =.57) but no association with emotional intelligence measures. IC-MD ratings were not related to high school performance or a cognitive student aptitude test, indicating divergent validity. Inter-rater reliability (*ICC* = 0.63) and generalizability (*Eρ*^*2*^ *=* 0.64) were satisfactory. The IC-MD proved to be fair regarding participants’ age and gender. Participants with prior work experience in healthcare outperformed those without such experience. Participant acceptance of the procedure were good. The IC-MD is a promising selection procedure capable of assessing interactional competencies relevant to the medical setting. Measures of interactional competencies can complement the use of cognitive selection criteria in medical student admission. The predictive validity of the IC-MD needs to be addressed in future studies.

## Introduction

Good doctors are well trained, informed, and knowledgeable about their specialist field, but also know how to utilize their interactional competencies to the benefit of their patients’ health and well-being. Based on research from the area of interactional video analysis, interactional competencies can be defined as contextual skills and abilities that individuals in social interactions use to engage in certain social practices and which are visible in the situation in which they are enacted (Biringen, 2000; Filliettaz et al., 2022; Young & Miller, 2004). Almost all stakeholders (healthcare personnel, patients, medical students, the general population, etc.) rank interactional competencies among the most important characteristics of a “good doctor” (Borracci et al., 2020; Steiner-Hofbauer et al., 2018). Interactional competencies are also known to be crucial for patient satisfaction, adherence to treatment, self-reported health-related quality of life, symptoms, and behaviours (Birkhäuer et al., 2017; Clever at al., 2008; Haskard Zolnierek & DiMatteo, 2009). Moreover, interactional competencies have been associated with fewer speciality care visits and hospitalizations and lower annual charges for medical care, consequently leading to reduced societal healthcare costs (Bertakis & Azari, 2011).

As such, interactional competencies can be viewed as just as crucial for health and societal outcomes as medical skills, and it therefore seems necessary to strengthen their role in medical student selection. Interactional competencies may complement the set of cognitive criteria in medical student selection procedures, ensuring that an applicant’s profile covers both interactional and cognitive requirements. Currently, however, most selection criteria used to regulate entry to German medical schools are aimed at cognitive performance, such as the high school grade point average (GPA) and aptitude tests, partly because non-cognitive criteria are typically harder to measure validly and/or lack conceptual clarity (Edwards et al., 1990; Streyffeler et al., 2005, Sommerfeld et al., 2011, Breil et al., 2022).

To capture non-cognitive admission criteria and overcome some of the known limitations of traditional admission interviews (e.g., reproducibility, generalizability), researchers at McMaster University in Canada developed Multiple Mini Interviews (MMIs) as a student admission procedure (Eva et al., 2004; Kreiter et al., 2004). In MMIs, applicants’ interactional competencies are evaluated in a circuit of multiple short and consecutive interview scenarios, providing a more structured approach and the possibility to observe candidates in multiple contexts and by multiple raters. MMIs can thus be classified as a simulation-based selection procedure. However, while MMIs have been found to be better predictors of academic performance than traditional interviews (Jerant et al., 2019), findings regarding their predictive validity remain mixed (e.g.Bußenius & Harendza, 2021; Husbands & Dowell, 2013; Knorr et al., 2018a; Kumar et al., 2018; Moll-Khosrawi et al., 2023). Moreover, the conceptualization of MMI stations (e.g. interviews, presentations, encounters with simulation patients) and the evaluation criteria differ widely across different medical schools.

### Challenges in the measurement of interactional competencies using MMIs

MMIs are not typically based on a theoretically and empirically sound conceptual framework. Instead, existing MMI procedures were usually developed based on considerations about requirements in the job field and according to expert consensus (e.g. Eva et al., 2004; Hissbach et al., 2014; Kumar et al., 2018). Specifically, experts compile a list of desired interactional competencies and a number of MMI scenarios are then devised, which are meant to capture these competencies. Such consensus-based approaches typically yield very similar lists of competencies, such as communication, empathy, or collaboration (Fürstenberg et al., 2017; Fürstenberg & Harendza, 2017; Hertel-Waszak et al., 2017; Lemay et al., 2007; Wijnen-Meijer et al., 2013). However, expert-consensus approaches (as opposed to construct-driven approaches) can give rise to a lack of conceptual clarity. For instance, there might be differences in the understanding of what empathy actually looks like in complex interactional situations such as those that occur in the medical field, and what it does or does not encompass. It is difficult to determine what conclusions about specific interactional competencies can be drawn from a given behavioural observation in a complex, quasi-realistic simulation. Accordingly, the construct validity of simulation-based approaches, including Assessment Centres (ACs) and MMIs, generally remains unclear, and it is argued that more research is needed to address this (Jackson et al., 2016; Knorr et al., 2018b).

Moreover, questions have been raised regarding whether variance in the measurement can actually be attributed to the desired construct(s) (Breil et al., 2022). For example, in a study by Breil et al. (2020), measurements of MMIs were not found to reflect the multiple interactional competencies in line with intention, but rather reflected general performance. This may be due to irrelevant factors that influence the measurement, as a number of observations of complex behavioural patterns are collected over various scenarios, possibly even with different formats (cf. Leduc et al., 2022) and by different raters, which may create error variance. Furthermore, it might not be easy to identify specific competencies on the basis of observations of complex behaviour in a quasi-realistic environment (Leduc et al., 2022; Lemay et al., 2007; Oliver et al., 2014). To address some of these issues, it may therefore be worthwhile to clearly define the measurement construct such that it (a) is readily observable under quasi-realistic conditions, (b) is conceptually clear, empirically well-founded, and sufficiently understood, and (c) reflects basic competencies in human communication that go beyond specific medical competencies.

In the context of assessing medical competencies, some authors have argued that it is necessary to target established psychological constructs rather than specific interactional competencies identified by medical experts. Indeed, in a quasi-realistic medical encounter, in which a complex interplay of medical knowledge and interactional competencies is needed to master any given situation, or in which there may not be any objectifiable phenomena at all, it might be impossible to capture specific expert-identified interactional competencies. Consequently, in order to test applicants in realistic settings, it is argued that the ideal of directly capturing lists of desirable interactional or medical competencies should be dispensed with (Lurie et al. 2012; Ten Cate & Scheele, 2007; van der Vleuten et al., 2010). In this work, we advocate for a different approach: If we can identify an established psychological construct, which is relevant to the medical context and has been empirically demonstrated to be observable and measurable in complex social interactions, it might be possible to avoid the usual construct validity issues associated with simulation-based approaches (cf. Breil et al., 2022 for a similar line of reasoning).

Within the medical selection and education context, interactional effectiveness has so far mainly been discussed as part of the more controversial psychological construct of *emotional intelligence* (Tiffin & Paton, 2020; Tiffin & Roberts, 2023). Emotional intelligence (EI) refers to individual abilities in perceiving, using, understanding, and managing emotions in interactional contexts (Mayer, 2004). Although research has demonstrated associations of EI with healthcare workers’ interactional and communicative abilities and with doctor-patient or nurse-patient relationship outcomes (Arora et al., 2010), due to its multidimensional nature, EI often proves to be a rather fuzzy construct, and might therefore be difficult to clearly operationalize and measure, especially in the medical context (Hellwig et al., 2020; Tiffin & Paton, 2020). Even if EI is measured in a performance-based manner rather than by direct self-report (the latter being prone to self-report bias, cf. Dulewicz & Higgs, 2000), evidence of the predictive validity of EI for selection purposes (i.e., prediction of academic success in medical school) is insufficient (Humphrey-Murto et al., 2014; Libbrecht et al., 2014). Importantly, measures of EI are not designed to capture performance in a realistic doctor-patient encounter, and may therefore be unable to account for a potential gap between knowledge and the ability to act accordingly in a contextualized social situation.

We therefore identified emotional availability (EA) as a construct with the potential to overcome these limitations (Biringen et al., 2019). EA is not only an established psychological construct, but can also be seen as a core construct that renders interaction within doctor-patient encounters effective. Notably, the theoretical and empirical findings within the field of EA research are based on observing the actual behaviours which manifest in dyadic interactions and are therefore directly and practically applicable to simulation-based approaches. The relevance of the concept of EA for a simulation-based medical selection tool such as an MMI is further corroborated by findings concerning practicability: A study on perceptions of simulated patients in medical education (Laughey et al., 2018) identified clearly observable cues for the effectiveness of interaction, similar to those established in the EA coding manual developed by the current authors as part of an MMI-based selection procedure at the Medical Faculty of the University of Heidelberg (e.g., open posture, fidgeting, or the avoidance of jargon).

### Emotional availability as a construct for assessing interactional competencies

EA encompasses a framework of interactional qualities that enable the formation of a stable and trusted relationship, originating from the concepts of attachment theory and emotion communication in the area of developmental psychology. The relevance of this framework for medical interactions is well established, with useful implications for different medical fields such as primary care and cancer care (Hooper et al., 2012; Kelly et al., 2019; Strauss, & Brenk-Franz, 2016). The use of attachment theory in the realm of medicine is also empirically supported by a substantial number of findings linking attachment security to physical health outcomes (Pietromonaco & Beck, 2019). For instance, attachment insecurity has been associated with higher symptom severity (Schroeter et al., 2015) and lower treatment success (Pfeifer et al., 2018).

The construct of EA was first conceptualized to evaluate observed interactions between caregivers and their children (Biringen, 2000). EA emphasizes the need for emotional attunement with and responsiveness to the emotional signals given by an interaction partner. As such, it may be considered as a prerequisite for “interpersonal effectiveness”, an aspect described by Tiffin and Roberts as part of their adapted model of emotional intelligence (EI) in relation to the ability to deliver person-centred care (2023). Biringen (Biringen et al., 2014) operationalized EA via the EA Scales, consisting of four important, though not distinct, components that promote the formation of a stable bond: sensitivity, structuring, non-intrusiveness, and non-hostility. The concept has already been broadened to describe interactions in adult care contexts (Cohen et al., 2022; Leinweber et al., 2019; Söderberg et al., 2014). For the purpose of an application to the doctor-patient relationship and utilization in medical student selection, the EA coding manual for caregiver-child relationships was adapted to characterize doctors’ communicative qualities in the clinical context (Biringen et al., 2019). Notably, the coding manual describes directly observable behaviours and relationship quality within the actual encounters.

*Sensitivity*. When seeing the doctor, patients are often in some kind of need or distress, which requires compassionate containing on the part of their conversation partner. A sensitive doctor needs to recognize and understand patients’ emotions, acknowledge their communicative signals, and compassionately guide them through difficult emotions, often despite a busy schedule that also requires them to elicit the necessary and relevant information within an extremely condensed time window. A lack of sensitivity can result in non-attendance of follow-up procedures or referrals, loss of trust, and the patient seeking help elsewhere (cf. Amelung et al., 2020).

*Structuring*. Due to the significant time constraints (Konrad et al., 2010), doctors need to structure conversations in an efficient way, ensuring that (a) they collect sufficient information from the patient and (b) the patient is provided with all relevant information, which forms an essential basis for shared decision making (Amelung et al., 2020). Furthermore, doctors need to combine efficiency with relationship-building, which includes helping patients to anticipate health trajectories and providing them with critical information in a time-sensitive manner.

*Non-intrusiveness*. Non-intrusiveness is a core concept of patient-centred care (cf. Deutscher Ärztetag, 2015) and requires doctors to encourage beneficial behaviour changes in their patients (e.g. a change in alcohol consumption or smoking, attendance of follow-up consultations) in such a way that patients’ autonomy is respected and time constraints are adhered to (cf. Dugdale et al., 1999; Parrish et al., 2016; Yahanda & Mozersky, 2020). Non-intrusive patient-centred care with respect for patients’ autonomy and boundaries also supports shared decision-making.

*Non-hostility*. Many of the characteristics of the clinical work environment, including time constraints, work overload, or “difficult” patient behaviours, can hamper doctors’ ability to express compassion (Fernando & Consedine, 2014; Malenfant et al., 2022). Thus, doctors need to regulate their own negative emotional responses in order to prevent them from interfering with the quality of their communication or their ability to openly address difficult topics. Hostile interactions between patients and therapists have been associated with stagnation or deterioration (Von Lippe et al., 2008), while missed opportunities to openly discuss the rationale for a procedure or referral may lead to confusion, mistrust, and disappointment on the part of the patient (Amelung et al., 2020).

### Present Study

In the present study, we explore whether a newly developed MMI-based selection procedure (*Interactional Competencies – Medical Doctors* (IC-MD; German: IKM) based on the concept of emotional availability meets the basic requirements for use as a reliable and valid instrument in medical student selection. More specifically, we seek to evaluate.

1. convergent validity (extent of associations between emotional availability as measured by the IC-MD and related (albeit not the same) constructs such as emotional intelligence,
2. divergent validity (lack of associations with cognitive admission criteria),
3. generalizability (the extent to which ratings generalize across the different scenarios),
4. inter-rater reliability (agreement between different raters in their evaluations of the observed interactions),
5. fairness (whether evaluations are independent of participants’ sociodemographic characteristics such as age and gender), and.
6. acceptance (how participants evaluate the procedure).

## Methods

### Procedure

Between 2019 and 2021, the MMI selection procedure and the coding manual for the IC-MD were continuously developed, evaluated, and improved based on data from three pilot cohorts of medical students. The data of the present study were obtained from the 2021 cohort. Assessments took place on two consecutive days in 2021 as part of a voluntary study conducted during the 1st month of undergraduate medical studies. Students participated in an MMI circuit consisting of five four-minute scenarios), which were each evaluated according to the same four subscales. The scenarios required, for example, participants to discuss health-preserving lifestyle changes with their patient or to resolve conflicts resulting from crowded waiting rooms. All scenarios were designed to pose a clear challenge in order to discriminate between different levels of participant performance. Participants were given three minutes between scenarios to move to the next scenario and read the next set of instructions, resulting in a total circuit time of 32 min. All scenarios were video-recorded. After each scenario, the simulation patients completed a short report of the perceived doctor-patient relationship. At the end of the circuit, participants completed additional measures on emotional intelligence (EI), sociodemographic characteristics, and on how they evaluated the procedure. The video recordings were coded by 17 raters (11 medical doctors, 5 psychologists, and 1 sociologist), who had received extensive training in the application of the IC-MD coding manual.

### Participants

Students at the beginning of their first semester of medical studies in 2021 were recruited via e-mail. To ensure that *N* = 70 students would participate in the MMI procedure even in the case of sickness or absence, we overrecruited one student for each of the circuits. Surplus students (*N* = 9) were only required to complete the questionnaires. All participants provided written informed consent.

### Measures

#### IC-MD

The IC-MD coding manual (Fuchs et al., 2021) consists of four subscales: Sensitivity, Structuring, Non-Intrusiveness, and Non-Hostility. Ratings on each subscale range from 1 (very problematic behaviour) to 7 (outstanding behaviour), with a score of 5 representing a typical “good enough” performance and a score of 4 representing inconsistent or inauthentic behaviour. Subscale scores are summed up to form a total score, the actual selection criterion resulting in a max. score of 28 for each MMI scenario. The manual provides detailed criteria on how to systematically evaluate the specific participant behaviours that are included in the respective subscales. In the present study, each video was coded by two independent raters, with each rater evaluating three different scenarios and a total of 42 videos, with the exception of one rater, who rated fewer videos.

#### Validation measures

As EI and EA are both multidimensional constructs that have been linked to interpersonal effectiveness and may therefore both be linked to doctors’ interactional and communicative abilities, we assume some theoretical and empirical overlap with our measure of interactional competencies in the doctor-patient relationship. However, as EI measures are designed to assess socio-communicative knowledge quite broadly, and often not within contexts relevant to medical student selection or in quasi-realistic settings, while the concept of EA is theoretically and empirically based on directly observed behavioural cues, we assumed no more than small to moderate associations with our measure.

##### TEIQue

The *Trait Emotional Intelligence Questionnaire* is a self-report measure of emotional intelligence (EI) (Petrides & Furnham, 2009), which covers four overarching factors: sociability (emotion management, assertiveness, social awareness), well-being (self-esteem, optimism, happiness), self-control (emotion regulation, stress management, low impulsiveness), and emotionality (empathy, emotion perception, emotion expression, relationships). Internal consistency of the total score is α = 0.74.

##### MSCEIT

The *Mayer-Salovey-Caruso Emotional Intelligence Test* (Mayer, 2002; Steinmayr et al., 2011) is a performance test measuring EI according to the areas of experiential EI (facets: perceiving emotion, using emotion) and strategic EI (facets: understanding emotions, managing emotions). Respondents are asked, for example, to judge emotional expressions presented in photographs (perceiving emotion) or to judge the appropriateness of emotions presented with the description of a situation (understanding emotion). Internal consistency of the four facets making up the total score amounts to α = 0.68.

##### PRA-D

The *Patient Reactions Assessment* questionnaire (Brenk-Franz et al., 2016; Galassi et al., 1992) was used to assess simulation patients’ evaluation of each consultation based on the informative, communicative, and affective qualities of the doctor-patient encounter. Internal consistency of the total score lies at α = 0.92.

##### Akzept!

The German-language Acceptance of Intelligence and Performance Tests questionnaire *(Akzept!*, Kersting, 2008) was used to assess participants’ perception of the selection procedure. The measure encompasses questions about perceived lack of burden, controllability, quality of measurement, and face validity. Items of the scales are rated from 1 (do not agree) to 6 (fully agree), with higher scores indicating greater acceptance.

##### Sociodemographic characteristics

To answer the research questions on fairness and convergent validity, participants were asked to indicate their age, sex, work experience in the healthcare system, student aptitude test score (Test für medizinische Studiengänge, TMS; English: Test for Medical Studies), and high school grade point average (GPA, possible range in the German school system from 1 – very good to 6 – inadequate).

### Data Analysis

For all data analyses, we applied an exploratory approach. Reliability analyses were performed using R Version 3.5.1. A generalizability analysis was performed including the facets *participant*, *scenario* and *rater* as well as the interactions *participant x scenario* and *rater x scenario* (see online supplement 1.1 and 1.2:: https://osf.io/t4yj8/?view_only=bb28d712f0f7464ca7738270d9b67562*)*. The inter-rater reliability was calculated using the different variance components (Var) as ICC = Var(*participant*) + Var(*participant x scenario*) / (Var(*participant*) + Var(*participant x scenario*) + Var(residual)). Generalizability for the IC-MD total score across the five scenarios was computed as Var(*participant*) / (Var(*participant*) + (Var(*participant x scenario*) + Var(residual))/5). Generalizability coefficients are presented as *Eρ*^*2*^, inter-rater reliability as intraclass correlation coefficients (ICC). For these mixed-model analyses, the R packages “lme4” and “lmerTest” were used.

All other data analyses were performed using Stata 16 (College Station, TX: StataCorp LLC). To obtain one score per IC-MD subscale per person, each participant’s performance was averaged across scenarios. A total score from the four subscales was calculated to reflect the overall IC-MD performance. Construct validity was established for the IC-MD total score.

Associations between IC-MD and continuous variables (MSCEIT, TeiQue, PRA-D, participant age, TMS, and GPA) are presented as Pearson’s correlation coefficients. Bonferroni-Holm corrections were applied to associations between the IC-MD total score and the MSCEIT, TeiQue, and PRA-D total scores. Associations between the IC-MD subscales are reported in an online repository (https://osf.io/t4yj8/?view_only=bb28d712f0f7464ca7738270d9b67562 cf. supplement 2, Table 1). According to Biringen (2000), the holistic judgement is of greater relevance; thus, single subscale scores are not further interpreted.

## Results

### Sample

*N* = 70 medical students with a mean age of M = 19.95 (SD = 2.20, range = 18–29) participated in the MMI. 57% were female, 90% spoke German as their first language, and 71% indicated that they had no more than six months of work experience in the healthcare system. However, the majority had completed shorter internships in the healthcare system (81%), as is very common among applicants to medical schools. Participants had a mean GPA of M = 1.22 (SD = 0.49, range = 1.0 to 3.4). 70% had taken the student aptitude test (TMS), achieving a mean standard score of M = 113 (SD = 7.20, range = 86 to 130). For *n* = 2 students, sociodemographic data are missing. A detailed sample description and descriptive data of the study variables are provided in Table 1.

**Table 1:** Sociodemographic data and descriptive data of study variables

Variable	Levels	*N* (%)
Sex	Female Male	39 (57.35) 29 (42.65)
First language	German Other	61 (89.71) 7 (10.29)
Parent works in healthcare system	Yes No	21 (30.88) 47 (69.12)
≥ 6 months experience in healthcare system	Yes No	20 (29.41) 48 (70.59)
Completed vocational training in healthcare system	Yes No	8 (11.76) 60 (88.24)
Internships in healthcare system	Yes No	55 (80.88) 13 (19.12)
Variable	M (SD)	Range
Age	19.95 (2.20)	18–29.67
TMS	113.77 (7.20)	86–130
GPA	1.22 (0.49)	1.0–3.4
*IC-MD*		
IC-MD total	21.32 (2.30)	15.00–25.85
Sensitivity	5.16 (0.63)	3.55–6.45
Structuring	5.23 (0.61)	3.70–6.45
Non-intrusiveness	5.31 (0.60)	3.65–6.45
Non-hostility	5.63 (0.56)	3.85–6.50
*MSCEIT*		
MSCEIT total	114.29 (13.75)	61–131
Perceiving emotion	105.44 (11.89)	73–122
Using emotion	113.76 (13.31)	69–131
Understanding emotion	110.33 (13.47)	64–129
Managing emotion	111.69 (13.76)	63–132
*TeiQue*		
TeiQue total	5.11 (0.54)	3.34–6.41
Sociability	5.03 (0.73)	2.98–6.43
Well-being	5.39 (0.89)	2.63–6.89
Emotionality	5.28 (0.73)	3.33–6.61
Self-control	4.74 (0.62)	3.44–5.97
*PRA-D*		
PRA-D total	15.63 (1.10)	12.78–18.54
Information	4.86 (0.47)	3.63–5.96
Communication	5.30 (0.41)	4.32–6.04
Emotional support	5.47 (0.58)	3.84–6.85

Note. *N* = 2 participants have missing data on the sociodemographic background questionnaire. TMS scores are available for *N* = 48 participants, as it is not a prerequisite for medical school admission

### Power analysis

Participant recruitment was restricted due to practical circumstances, resulting in a relatively small sample size. We conducted a power analysis to determine the increase in sample size which would have been required to detect a medium effect of *r* =.3 with a power of 0.72 within our sample of *n* = 70, with a power of 0.80. The minimum sample size would have been *n* = 84.

### Construct validity: main analyses

*Convergent validity*: Overall, the evaluation of the IC-MD performance showed a strong significant association with simulation patients’ judgement of the consultation (PRA-D, *r* =.57, *p*^adjust^ < 0.001). The IC-MD total score was not significantly associated with the EI performance test (MSCEIT, *r* =.25, *p*^adjust^ = 0.072) or with the EI self-report (TeiQue, *r* =.19, *p*^adjust^ = 0.110). The subscale results revealed comparable findings (cf. online supplement 2, Table 2, https://osf.io/t4yj8/?view_only=bb28d712f0f7464ca7738270d9b67562).

### Divergent validity

IC-MD total scores were not significantly associated with cognitive ability as assessed by the TMS (*r* =.01, *p* =.967) or with high school GPA (*r* =.14, *p* =.316) (for an overview, see Table 2).

**Table 2:** Construct validity: Correlations between IC-MD total and validation constructs (Pearson’s r)

	Total IC-MD		
Convergent validity	*R*	*p*	*p* ^*adjust*^
MSCEIT total	0.25	0.036	0.072
TeiQue total	0.19	0.110	0.110
PRA-D total	0.57	< 0.001	< 0.001
**Divergent validity**			
TMS	0.01 (0.01)^a^	0.967	
GPA	0.14 (0.12)^a^	0.316	

Note. *p*^adjust^ = Bonferroni-Holm-corrected *p*-values. ^a^ Correlations between IC-MD and TMS/GPA are corrected for range restriction (using Thorndike´s Case II formula) due to the pre-selection via TMS/GPA. Values in brackets are not corrected for range restriction

### Generalizability and inter-rater reliability

The amount of variance explained by *participant x scenario* was approximately 1.4 times higher than the variance explained by *participant* only (see Table 3 for a full overview). Intercorrelations between stations were small to medium, and can be found in the online supplement (supplement 2, Table 3https://osf.io/t4yj8/?view_only=bb28d712f0f7464ca7738270d9b67562*)*. Associations between the IC-MD subscales and IC-MD stations with the different constructs EI and PRAD-D are also presented in the supplement (cf. online supplement 2, Tables 2 and 4https://osf.io/t4yj8/?view_only=bb28d712f0f7464ca7738270d9b67562*)*. For the IC-MD total score, the generalizability was *Eρ²* = 0.642 and the inter-rater reliability was ICC = 0.627 (see Table 4). To achieve a generalizability value of ≥ 0.80, 12 scenarios would have been necessary. A detailed description of the generalizability analysis is provided in the online supplement (https://osf.io/t4yj8/?view_only=bb28d712f0f7464ca7738270d9b67562, supplements 1.1 and 1.2). 

**Table 3:** Variance component estimates for the facets participant, scenario, and rater

	Variance	Standard Deviation
Participant	3.75	1.94
Rater	2.04	1.43
Scenario	1.02	1.01
Participant x scenario	5.16	2.27
Rater x scenario	0.50	0.71
Residual	5.30	2.30

Note. In addition to the measurement error term, residual variance also entails Var(participant x rater) as well as a threefold interaction term Var(participant x rater x scenario), as the two variance components could not be separated from measurement error

**Table 4:** Reliability (generalizability and inter-rater reliability) of IC-MD over the 5 scenarios

	Eρ^2^ (standard error)	ICC (standard error)
Total IC-MD	0.642 (0.059)	0.627 (0.035)
Sensitivity	0.634 (0.067)	0.600 (0.036)
Structuring	0.625 (0.067)	0.586 (0.045)
Non-intrusiveness	0.598 (0.067)	0.559 (0.042)
Non-hostility	0.569 (0.072)	0.531 (0.044)

### Fairness

IC-MD total scores were not significantly associated with participants´ age (*r* =.10, *p* =.433) and did not significantly differ between male and female participants (*t(*66) = -1.30, *p* =.196). Participants with German as their first language (*M* = 21.56, *SD* = 2.27) achieved a significantly higher IC-MD total score than did participants with any other first language (*M* = 19.2, *SD* = 1.79; *t*(66) = 2.65, *p* =.010). Participants who had worked in the healthcare system for ≥ 6 months before entering medical school (some of them having received vocational training) significantly outperformed those without professional experience in the healthcare system (experience of ≥ 6 months: *M* = 22.78, *SD* = 1.84; less or no experience: *M* = 20.71, *SD* = 2.25; *t*(66) = 3.63, *p* =.001).

#### Acceptance

Participants’ mean acceptance scores ranged from 3.9 (quality of measurement) to 5.4 (controllability), with 6 indicating “full agreement” (see Table 5). Participants also provided feedback about their general perception of the IC-MD procedure as well as an estimate of their own performance compared to other participants in the form of school grades (1 = very good; 6 = inadequate). Participants predominantly provided positive general feedback on the IC-MD procedure, with an average score of 1.8 (good, possible range from 1 (most positive) to 6 (most negative)) and believed their own performance to be good to satisfactory on average (2.57).

**Table 5:** Descriptive statistics of the applicant reactions questionnaire: Participants’ judgement of the IC-MD procedure

Variable	M	SD	Min	Max
Controllability	5.40	0.60	3.5	6.0
Quality of measurement	3.93	0.73	2.0	6.0
Face validity	4.83	0.88	2.5	6.0
Perceived lack of burden	4.74	0.97	1.75	6.0
Overall perception of the procedure*	1.79	0.66	1.0	4.0
Self-rated performance*	2.57	0.84	1.0	5.0

* Overall perception of the procedure and self-rated performance were rated as a school-grading scale where lower values indicate a more positive judgement

For controllability, quality of measurement, face validity, and freedom of burden, higher values indicate a more positive judgement.

## Discussion

To explore whether the empirically and theoretically well-founded concept of emotional availability can be used as a basis to adequately assess the interactional competencies of medical school applicants in a quasi-realistic setting, we adapted an EA manual to the doctor-patient context for use in a newly developed MMI-based selection procedure (IC-MD). The IC-MD was shown to be meaningfully associated with simulation patients’ perceptions of the interaction but did not correlate significantly with self-reported and performance-based measures of EI after correction for multiple testing. Fairness in terms of age and gender was substantiated and sufficient inter-rater reliability and generalizability were confirmed. Medical students in their first month of studies evaluated the new procedure positively, as revealed by measures of participant reactions. Moreover, performance in the simulation-based IC-MD was independent of results from the major cognitive selection criteria of TMS and GPA scores.

The conceptualization of our measure as a theory-based approach might set the IC-MD apart from earlier, typically consensus-based approaches to rating MMI performance. We aimed to avoid the shortcomings of these approaches, which may typically interfere with an instrument’s construct validity. In the IC-MD, a substantial amount of variance in the students’ interactional competencies was explained by the range of participants’ scores. However, we also found that a higher proportion of variance was attributed to the interaction between participants’ performance and scenarios than to participants’ performance alone. The correlations between scenarios, although significant, were small to medium only, indicating that there is still variance between scenarios that we are unable to explain. This needs to be further addressed in a study with more participants and higher power. At the same time, associations among the subscales of EA within the scenarios were high, further corroborating Biringen’s notion that the subscales represent related constructs that assist in providing a holistic impression of participants’ EA (Biringen, 2000) and supporting our approach to use only the total score for further analyses and for selection purposes.

We believe that it is worthwhile to seek to avoid ambiguities in measurement and thus circumvent validity issues arising from the fuzziness and broad scope of concepts such as EI. Within the model of EI adapted to the medical context by Tiffin and Roberts (2023), our concept of EA can be assumed to fall under some of the listed constructs that make for effective interaction. That is, several of the precursors to the construct of “interpersonal effectiveness” in their adapted model may be related or similar to aspects of EA. For example, “emotional stability”, as postulated in the model, might be seen as a prerequisite for exhibiting non-hostile behaviour, with non-hostility being a subscale of EA. However, we assume that based on the research tradition of EA, which is rooted in direct observation, it may be more useful in a simulated behavioural assessment setting such as an MMI.

Importantly, due to the slightly underpowered nature of our sample size, the present results need to be interpreted with caution. However, the absence of significant correlations between the IC-MD and measures of EI in our sample may still indicate that a large overlap is not to be expected and larger effect sizes are unlikely, as EI and EA are distinct concepts stemming from very different research traditions: The concept of EA is rooted in the direct observation of behavioural cues in dyadic interactions, making it highly suitable for a simulation-based approach such as an MMI, while EI is based on a more theoretical tradition of assessment by self-report, similar to the construct of intelligence, which may be more difficult to adapt to quasi-realistic settings. Previous research similarly did not find substantial associations between observation-based MMIs and either self-report or performance-based EI measures (Knorr et al., 2018a; Yen et al., 2011). As pointed out in the introduction, medical student selection should aim at identifying candidates who are suited for both the cognitive demands of the curriculum and profession and the interactional demands that come with a clinician’s tasks. The lack of correlation of the IC-MD with TMS and GPA scores, along with the variance in the study participants’ interactional competencies, indicate that adding the IC-MD to existing cognitive criteria may yield additional value in identifying ideal candidates, although we cannot yet make any claims about the procedure’s incremental prognostic validity. The generalizability of 0.64 for the total score across scenarios fits well within the range of generalizability values reported in review articles on MMI studies, lying between approx. 0.50 and 0.88 (Knorr & Hissbach, 2014; Pau et al., 2013), and can thus be considered desirable for an MMI procedure consisting of just five scenarios. Contrary to other MMIs, which were designed to measure one individual interactional competency within each individual scenario (Pau et al., 2013; Oliver et al., 2014), we aimed to reliably measure the broad concept of EA by aggregating its subscales across the different scenarios. Nevertheless, we are continuously reviewing individual scenarios, and have, for example, already replaced one of them due to its low level of difficulty compared to the others. This requires the continuous testing of generalizability in light of new developments.

At 0.63, inter-rater reliability fell within the (albeit lower) range of IRR scores (0.52 to 0.91) reported in other MMI studies (Knorr & Hissbach, 2014). However, following the 2021 pilot, some additions have been made to the training of the raters to further ensure and improve the quality of the selection procedure. To guide raters through the reliability process, gold standard video ratings are now used. These were developed for training videos in an iterative consensus-based approach by the two trainers who were also involved in the original adaptation of the manual to the medical context (IS, AF). Raters need to achieve IRR scores with the gold standard of at least 0.7 before they can become certified as raters. In contrast to our study, several other studies found that female candidates receive higher scores on MMIs than male candidates (e.g. Jerant et al., 2015; Knorr et al., 2019). It should be mentioned, however, that due to considerably larger sample sizes, these studies had greater statistical power to detect group differences. Moreover, some previous studies found that older participants tended to perform better in MMIs (Jerant et al., 2015; Knorr et al., 2018b; Leduc et al., 2017). Given the very small effect size and its lack of statistical significance in the present study, age seems to be largely irrelevant for the IC-MD, thus emphasizing the fairness of the procedure. This lack of an age effect also shows that the positive impact of prior work experience in the medical field is not due to general life experience, but rather to contextualised experience. In line with other MMI procedures, however, the status of being a native German speaker significantly impacted IC-MD performance (Kelly et al., 2014; Knorr et al., 2019; Leduc et al., 2017). The fact that non-native speakers achieved lower scores – even though a language proficiency certificate is needed for admission to German medical schools – implies that the interactional competencies measured in MMIs such as the IC-MD rely on language proficiency. While empathy and compassion can also be expressed nonverbally, many challenging consultations require a substantial degree of nuance in the use of language. Additionally, the increased mental load due to communication in another language may leave less capacity for emotional undertones.

Follow-up studies on predictive validity are needed to indicate whether we are able to measure EA using the IC-MD procedure with sufficient reliability to predict study success in exams that combine medical knowledge and interactional competencies, such as the Objective Structured Clinical Evaluation (OSCE). Interestingly, participants who had work experience in the healthcare system performed significantly better on the IC-MD. Evidence indicates favourable effects of communication training in medical doctors (Mata et al., 2021), and medical school curricula incorporate communication training with the expectation that this will better prepare students for patient contact (Rheingans et al., 2019; Schultz et al., 2007). Work experience with (challenging) patient encounters might similarly train interactional competencies. Thus, applicants who have work experience or vocational training in the healthcare system might have an advantage in the IC-MD. Nevertheless, vocational training alone might not be a sufficient selection criterion, as the available evidence for its predictive validity is mixed (Hampe et al. 2009; Amelung et al., 2022). Our procedure allows us to directly identify talented candidates independent of prior experience. It is important to note that experience does not always go hand in hand with an improvement of interactional competencies. For instance, in a longitudinal study of medical students, Hojat et al. (2009) showed that there was a significant decline in empathy in the third year of medical school, the year in which patient care becomes a more central part of the curriculum. Critically, this decline was especially steep in students with lower baseline empathy, underlining the importance of selecting candidates who exhibit particularly high degrees of compassionate behaviour from the outset. Since time pressure, lack of role models, and a hostile work environment were mentioned as reasons for this decline, there is a risk that structural issues of the working environment may hamper emotional availability and patient-centred care. Therefore, following Tiffin and Roberts (2023), it seems crucial to select candidates who are able to manage the challenges of everyday medical practice and time pressure well. In line with this, the IC-MD ratings explicitly include participant behaviour in the context of stress and criticism, reflecting candidates’ ability to regulate their own emotions and behaviour under pressure. As selection for medical schools is highly competitive, a combination of criteria reflecting both social and cognitive abilities seems reasonable in order to select the most suitable candidates.

### Limitations and future directions

The current study is exploratory in nature and employed a cross-sectional design, and we do not yet have any data on how the 70 participants in our study will perform during exams requiring interactional competencies (e.g., OSCE) and how the IC-MD will predict behaviour in the clinical setting. A follow-up of the pilot cohorts is ongoing and we plan to further validate the IC-MD by examining the study success of those students who were selected via the IC-MD procedure in the upcoming years and even beyond their studies, in the first years of professional life. A larger pool of possible scenarios, e.g. also including cultural competence in medical care (Tiffin & Roberts, 2023), will be developed and we will investigate whether generalizability can also be assumed and even increased across these new scenarios. Lastly, the present study was slightly underpowered. With additional cohorts added to the analyses, we expect to be able to sufficiently increase sample size and consequently power in order to corroborate our findings.

## Conclusions

Doctors’ interactional competencies play a major role in patient well-being. The newly developed medical school selection procedure IC-MD allows for the assessment of interactional competencies based on the theoretical and well-founded construct of emotional availability, potentially overcoming the typical construct validity ambiguities associated with simulation-based approaches. It comprises the behavioural qualities necessary for the development of a trustful doctor-patient relationship in the challenging context of a clinical setting. Despite the slightly underpowered sample size, initial evidence for construct validity is provided by associations with simulation patients’ perception of the consultation, supporting the procedure’s convergent validity, and the lack of correlation with the GPA and the student aptitude test TMS, supporting the divergent validity of the IC-MD. Measures of interactional competencies should be used as selection tools to complement traditional (cognitive) selection criteria, reflecting the ideal profile of a medical school candidate. Future investigations need to reveal how IC-MD performance predicts academic success and interactional competencies in actual clinical settings.

## Data Availability

The data that support the findings of this study are not openly available due to reasons of consent.
